# Higher Vulnerability and Stress Sensitivity of Neuronal Precursor Cells Carrying an Alpha-Synuclein Gene Triplication

**DOI:** 10.1371/journal.pone.0112413

**Published:** 2014-11-12

**Authors:** Adrian Flierl, Luís M. A. Oliveira, Lisandro J. Falomir-Lockhart, Sally K. Mak, Jayne Hesley, Frank Soldner, Donna J. Arndt-Jovin, Rudolf Jaenisch, J. William Langston, Thomas M. Jovin, Birgitt Schüle

**Affiliations:** 1 The Parkinson’s Institute, Sunnyvale, California, United States of America; 2 Max Planck Institute for Biophysical Chemistry, Göttingen, Germany; 3 Molecular Devices, LLC, Sunnyvale, California, United States of America; 4 The Whitehead Institute and Department of Biology, MIT, Cambridge, Massachusetts, United States of America; UCL Institute of Neurology, United Kingdom

## Abstract

Parkinson disease (PD) is a multi-factorial neurodegenerative disorder with loss of dopaminergic neurons in the *substantia nigra* and characteristic intracellular inclusions, called Lewy bodies. Genetic predisposition, such as point mutations and copy number variants of the *SNCA* gene locus can cause very similar PD-like neurodegeneration. The impact of altered α-synuclein protein expression on integrity and developmental potential of neuronal stem cells is largely unexplored, but may have wide ranging implications for PD manifestation and disease progression. Here, we investigated if induced pluripotent stem cell-derived neuronal precursor cells (NPCs) from a patient with Parkinson’s disease carrying a genomic triplication of the SNCA gene (SNCA-Tri). Our goal was to determine if these cells these neuronal precursor cells already display pathological changes and impaired cellular function that would likely predispose them when differentiated to neurodegeneration. To achieve this aim, we assessed viability and cellular physiology in human SNCA-Tri NPCs both under normal and environmentally stressed conditions to model *in vitro* gene-environment interactions which may play a role in the initiation and progression of PD. Human SNCA-Tri NPCs displayed overall normal cellular and mitochondrial morphology, but showed substantial changes in growth, viability, cellular energy metabolism and stress resistance especially when challenged by starvation or toxicant challenge. Knockdown of α-synuclein in the SNCA-Tri NPCs by stably expressed short hairpin RNA (shRNA) resulted in reversal of the observed phenotypic changes. These data show for the first time that genetic alterations such as the *SNCA* gene triplication set the stage for decreased developmental fitness, accelerated aging, and increased neuronal cell loss. The observation of this “stem cell pathology” could have a great impact on both quality and quantity of neuronal networks and could provide a powerful new tool for development of neuroprotective strategies for PD.

## Introduction

Two of the most critical parameters determining cellular functionality and health are energy generation and distribution, because they are the driving force behind all biological processes [1]. Mitochondria are at the center of cellular metabolism and energy-dependent signaling processes [2], compartmentalizing cellular bioenergetic pathways and linking cellular energy to gene expression [3]. Thus, mitochondria are directly involved in regulation of cell fate and neuroplasticity.

In the process of neuronal degeneration, mitochondria are central regulators of cellular fate and apoptosis [4]. It is not surprising then that impaired mitochondrial function has been shown in several neurodegenerative disorders, including Parkinson’s disease (PD). Interestingly, mitochondrial dysfunction has been implicated in various inherited forms of parkinsonism as well (e.g. *Parkin*, *PINK1*, *LRRK2*, and *SNCA* genes) [5]. Apart from gene defects, environmental toxicants directly affecting mitochondrial function have been identified as risk factors for PD etiology [6].

The α-synuclein (*SNCA)* gene was the first gene linked to familial PD [7], and genetic alterations at the SNCA locus are one of the most significant genetic risk factors for development of sporadic forms of the disease [4], [8], [9]. The *SNCA* triplication (SNCA-Tri), resulting in an overexpression of wildtype α-synuclein protein (α-syn) which leads to early onset progressive parkinsonism, represents an ideal system to investigate synucleinopathy-specific disease mechanisms [10], [11].

α-syn is associated with mitochondria [12], and pathological α-syn oligomers at mitochondrial membranes exhibit direct mitochondrial toxicity [13], [14], affect mitochondrial dynamics [15], [16] and interaction with other organelles [17]. In skin fibroblasts from the patient described in this study, we found significant changes in ATP production, reduction in mitochondrial membrane potential and complex I activity. The SNCA-tri fibroblasts were also more sensitive to oxidative stressors. The phenotype could be partially reversed by siRNA knockdown of α-syn which suggests a direct causative role for increase concentrations of intracellular of a-syn [18].

Mitochondrial integrity and functional metabolism are also essential for stem cell proliferation [19], [20] as well as differentiation [21]–[23]. Until now, cell biology and metabolic properties of human neural stem cells carrying PD mutations have not been thoroughly investigated, and even less is known about the impact of environmental factors on these cells.

Generating induced pluripotent stem cells (iPSCs) from patients with PD and deriving differentiated progeny with PD-specific phenotypes [24]–[27], now allows for *in vitro* modeling and investigation of disease mechanisms at different developmental stages [27]–[34].

In this study we take advantage of this new technology to investigate the impact of excess intracellular concentrations of a-syn (using tissue from our patient with a *SNCA* gene triplication (SNCA-Tri)) on cellular and mitochondrial function of human iPSC-derived neuronal precursor cells (NPCs). Using semi-quantitative and high throughput screening (HTS) and high content imaging (HCI) technologies in addition to biochemical assays and conventional imaging, we observed that there are profound effects on mitochondria function and energy production in this novel model of PD. As iPSC-derived NPC populations can give rise to a variety of neuronal cell types, our studies may warrant further investigations on how decreased developmental fitness, accelerated aging and increased susceptibility to stress contribute to neurodegenerative processes that set the stage for to neuronal cell loss in PD.

## Materials and Methods

### Generation of patient-derived induced pluripotent stem cells (iPSC)

We generated multiple iPSC clones from fibroblasts from a patient with parkinsonism carrying a *SNCA* triplication and a four year older mutation-negative healthy female sibling [24]. Two iPSC clones with normal karyotype were used for further analyses. As an additional control, an iPSC line from a healthy age and sex-matched mutation-negative individual was generated. The study (protocol ECH-08-20) was approved by a local ethics committee (El Camino Hospital, Mountain View, CA), reviewed annually, and all participants signed informed consent.

### NPC derivation and propagation

We used our published protocol for the generation of NPCs by neuronal induction from embryoid bodies combined with dual SMAD inhibition and PSA-NCAM magnetic-bead sorting [35]. Two iPSC-derived NPC clones from the SNCA-Triplication patient (clones 1754-MIT; 1754-C7) were used for this study. In addition to the sibling control (clone 1761-C1) the NPC clone from a normal control (clone 1815-17-21) was used as an unrelated control cell line. Briefly, NPCs (passage15–30) were seeded at 5×10^3^ cells/mm^2^ on 300 µg/ml Geltrex (Life Techn. #A10480-02) and propagated in NPC growth medium (Life Technologies: Neurobasal medium #21103; 2mM L-Glutamine #25030; 1X NEAA #111400; 1X B27 #17504-044; and R&D systems: 20 ng/ml bFGF2 #PHG-0263) for up to 15 passages. Near confluent NPC cultures in 6-well plates were passaged with Accutase (Innovative Cell Technologies #AT104).

### SNCA small hairpin RNA (shRNA) NPC line

To investigate potential phenotypical rescue by lentiviral SNCA gene knock-down, the vector pLKO.1 puro (Sigma Aldrich #SHC001) expressing the 5′-CCGGACCAAAGAGCAAGTGACAAATCTCGAGATTTGTCACTTGCTCTTTGGTTTTTT-3′ (clone ID: TRCN0000003736) was used to knock-down human aSyn gene in NPCs. All cloned sequences were verified by automated sequencing (StartSeq, Mainz, Germany). Lentivirus infected cells were selected using puromycin.

### NPC toxicant and inhibitor treatment

For standard microscopy, NPCs were seeded at 1×10^4^ cells/mm^2^ on poly-ornithine/laminin (20 µg/ml/0.5 µg/ml, Sigma, #P4957, #L2020) coated 13 mm coverslips (Thermo Fisher, #10252961) or Lab-Tek chamber slides (Thermo Fisher Nunc #177445) the day before the experiment. Similarly, 24-well plates for flow cytometry experiments were seeded at 2×10^4^ cells/mm^2^ and 96-well plates (Greiner Cellcoat #655936, Perkin Elmer ViewPlate #6005225) were seeded at 1×10^4^ cells/mm^2^ for HCI microscopy and 2×10^4^ cells/mm^2^ for HTS plate reader analysis, biochemistry and ELISA assays.

For experiments with toxicants and inhibitors, NPCs were cultured under the following conditions for 18 hrs unless specified differently in the experimental procedures:

Standard NPC growth medium as above (HG = high glucose), HG plus 20 µM rotenone (Sigma #R8875) (HG+R) or modified NPC growth medium without glucose (NG) (Life Techn. Neurobasal-A, Formula 05-0128DJ). For experiments determining Reactive oxygen species **(**ROS) generation, B27 was replaced with the formulation without antioxidants (Life Tech, #1889-038).

Cells were treated for 1 hr before measurement with the following ROS generators, apoptosis inducers and mitochondrial inhibitors: 200 µM tert-butyl-hydroxy-peroxide (TBHP) (Sigma, #458139) for 1 hr, 100 µM paraquat (PQ) (Fluka, #36541), 2 µM oligomycin (O) (Sigma, #O4876) for 2 hrs, 1 µM ionomycin (Iono) (Sigma #I0634) or 2 µM CCCP (Sigma, #C2759) for 1 hr.

Inducers of mitochondrial apoptosis: 4 µM staurosporine (SP) (Sigma, S5921) for 1 or 4 hrs. Inhibitors of mitochondrial apoptosis: 1 µM cyclosporine A (CsA) (Sigma #C3662) for 12 hrs. Inhibitor of the multi-drug resistance transporter: 25 µM cyclosporine D (CsD) (SCBT, #sc-204702). All inducers and inhibitors were from concentrated stock solutions prepared in sterile DMSO or H_2_O.

### Immunocytochemistry (ICC)

NPCs were fixed in 4% PFA (EMS, #RT15700) for 10 min at room temperature (RT). Cells were permeabilized with 0.3% Triton X-100 in PBS for 5 min, washed with PBS, blocked with 5% goat serum (Vector Labs #S1000) for 30 min at RT, and incubated with primary antibodies in 3% goat serum for 2 hrs at RT. Indirect immunofluorescence staining was performed with (Life Techn., Alexa-350, -488 and -594 conjugated H+L antibodies #A11059, #A11046, #11011). Cells were mounted with Vectashield (Vector Labs #1400). For anti-α-syn ICC, cells were fixed as above, blocked with PBS, 3% BSA, 0.1% Triton X-100 for 1 hr and incubated in blocking solution containing the polyclonal anti-α-syn antibody (Millipore AB5038, 1/100) at 4°C over night. After three washes in PBS, 1% BSA, 0.1% Triton X-100, α-syn was detected using the above Alexa-488 conjugated secondary antibody for 1 hr at RT. After three washes in PBS, cells were mounted as described above.

### Protein analysis

1×10^6^ NPCs (passages 19, 23 and 27 were lysed on ice in 250 µl 1x RIPA buffer (Sigma #R0278). Protein concentration was determined with a BCA Assay (Pierce #23235). 30 µg protein was resolved by SDS-PAGE on NuPAGE 4–12% Bis-Tris pre-cast gels (Life Tech.), transferred onto Immobilon-FL 0.45 µm PVDF membranes (Millipore, #IPFL10100) and detected with the Li-COR Odyssey Western blotting Kit I or II (LI-COR Biosciences P/N 926-31081/2). Immuno-reactive bands were detected and quantified with the LI-COR ODYSSEY infrared imager. The polyclonal anti-α-syn specific antibody (1/300) was from Millipore (#AB5334P). HSP90 (#4874), β-tubulin (#2146) at and cytochrome c (#4272) all used at 1/1000 were from Cell Signaling Technologies.

### Cell viability and survival

NPCs on Geltrex-coated 96-well plates were challenged as described under method section: NPC toxicant and inhibitor treatment, with the exception that 20 µM paraquat was applied. Medium was changed daily and cells were imaged with Zeiss Axiovert 25 microscope (with CP-Achromat 10x/0.25 Ph1 objective) and Canon EOS400 camera. Images were contrast enhanced, converted to grayscale and number of cells per image frame was analyzed with ImageJ [36].

### High content imaging (HCI) and high throughput screening (HTS)

Conventional fluorescence microscopy (Nikon Eclipse T*i*, Nikon Planfluor Objectives 10x/.03, 40x/0.75, 60x ELWD/0.7; Chroma 4900 series filtersets: ET-DAPI, -GFP/FITC, -CY3, -mCherry/Texas Red) was confirmed/validated by high throughput/content screening.

### Plate reader HTS

For HTS plate reader analyses, several independent experiments (as specified in results) with 3–4 replicates per cell line and treatment regimen were conducted. Signals from 96-well plates (integration time 1 sec) were acquired by bottom read in orbital mode (8-spot measurements with 2 mm radius). Kinetic measurements were recorded over 30 min with a sampling rate of 1 read/min. Data analysis was performed with BMG Mars software.

### HCI microscopy

HCI microscopy was performed with an ImageXpress Micro System, Molecular Devices LLC and HTS was performed with a multi-wavelength plate reader (Polarstar Omega, BMG) or by flow cytometry (Accuri C6 with C-sampler, BD Biosciences). Images from NPCs seeded in 96-well plates were acquired with 10x and 20x Plan Fluor objectives (4 replicates with 4 sites per well) using Ex./Em. 346/480 nm, 490/530 nm and 535/585 nm filter sets and then analyzed with the MetaXpress software and the following modules: Cell Scoring (cell metrics), Granularity (endpoint analysis of organelle and mitochondrial metrics) and Transfluor (co-localization of two or more fluorophores).

### Mitochondrial membrane potential (MMP)

Adherent NPCs in 96-well plates were washed twice with 200 ul HBSS, incubated with 100 nM tetra-methyl-rhodamine methyl ester (TMRM, Ex./Em. 535/580 nm) (Life Techn. #T-668) or with 20 µM of the ratiomeric MMP probe JC10 (with 0.02% Pluronic F-127, Life Techn. #P6867) in HBSS^plus^ (HBSS #14025-126, 2 mM L-glutamine #25030-081, 100 µM Na-pyruvate #11360-070, all Life Techn.; 100 µM Na-succinate, #S9637 SIGMA) for 45 min under standard growth conditions. Five min before analysis, 1 µM Hoechst 33342 (Ex./Em. 350/461 nm) (Life Techn. #H3570) was added. Cells were washed twice and covered with 100 µl HBSS^plus^ for fluorescence acquisition at Ex./Em. 544/590±10 nm for TMRM, Ex./Em. 544/590±10 nm and 488/535 nm for JC10, 355/460 nm for Hoechst 33342. TMRM fluorescence intensities or JC10fluorescence ratios were normalized to the Hoechst 33342 signal.

### Reactive oxygen species (ROS)

Adherent NPCs in 96-well plates were washed twice with HBSS and loaded with 15 µM 5-(and-6)-chloromethyl-2′,7′-dichlorodihydrofluorescein diacetate, acetyl ester (CM-H_2_DCFDA, Ex./Em. 495/520 nm, Life Techn. #C6827) under in HBSS^plus^ for 30 min at 37°C with 1 µM Hoechst 33342 and 100 nM MitoTracker CMXRos (Life Techn. M7512 Ex./Em. 579/599 nm). After two washes in HBSS^plus^ fluorescence signals for CM-H_2_DCFDA at Ex./Em. 485/520 nm, MitoTracker at 544/590±10 nm and Hoechst 33342 were acquired. Relative CM-H_2_DCFDA fluorescence intensities were normalized to MitoTracker and Hoechst 33342.

### Mitochondrial superoxide

Adherent NPCs in 96-well plates were incubated with 2 µM MitoSOX (Ex./Em. 510/580 nm) (Life Techn. #M36008) in HBSS^plus^ for 10 min at 37°C in the dark. Cells were then washed, MitoSOX fluorescence was detected at Ex./Em. 544/590±10 nm and normalized to cell number as under method section: ROS.

### Mitochondrial permeability transition pore (MTP)

Adherent NPCs in 96-well plates were loaded for 15 min with 1 µM calcein AM (Life Techn. #C3099, Ex./Em. 494/517 nm), 1 µM CoCl_2_ (Sigma #C2644) in HBSS^plus^ with Mitotracker and Hoechst 33342 as described under “ROS”. Cells were treated for 1 hr with either 700 nM TBHP or with 4 µM SP and analyzed as described under section ROS, with calcein fluorescence acquired at Ex/Em. 485/520 nm. 2 µM ionomycin (Iono) was used as negative control.

### Caspase activation

Caspase activity was assayed from NPCs seeded in 96-well plates with the EnzChek Caspase-3 Assay Kit (Life Techn. #E-13183). Apoptosis was induced with 4 µM SP for 4 hrs. After cell lysis, an aliquot was removed for protein (BCA-Assay) and 20 µM Z-DEVD–AMC substrate (Ex./Em. 342/441 nm) was added. After 30 min incubation at RT, fluorescence of the cleaved Z-DEVD–AMC substrate was acquired (Ex./Em. 380/445 nm) and normalized to AMC standard and protein content. Change in caspase activity (Δ µM AMC/mg protein) in NPCs was measured by detaching cells from substrate and permeabilization with 50 µg/ml digitonin (Sigma, #D5628) in HBSS^plus^ for 10 min at RT. After addition of caspase substrate, fluorescence was recorded every 30 min for 4 hrs.

### Metabolic flux analysis

Cellular response to specific mitochondrial inhibitors was analyzed on a Seahorse XF24 by monitoring O_2_ consumption (OCR) and extracellular acidification rates (ECAR). Three days before the assay 1×10^5^ cells were seeded on V7 24-well plates (Seahorse Bioscience, #SEA100777004) pre-coated with 150 µg/ml Geltrex. Culture medium was changed to Seahorse Assay Medium (#SEA102353100) supplemented with 10 mM pyruvate and 25 mM glucose one hour before starting the measurements. The neurotoxin 6-hydroxydopamine (6-OHDA) at 250 µM final concentration (f.c.) was added to half of the wells. OCR and ECAR baselines were recorded three times before the consecutive addition of (all f.c.): 1 µM oligomycin, 1.5 µM CCCP and 5 µM rotenone (Rot) also containing 1 µM antimycin A (Ant). Control wells without addition of drugs were recorded in every plate as a viability reference for each cell line and used to correct the values of spare respiratory capacity (OCR_CCCP_–OCR_basal_) and proton leakage (OCR_Oligomycin_–OCR_Rot+Ant_).

### Flow cytometry

NPCs were dissociated with Accutase and resuspended in PBS at 1–2×10^6^ cells/ml. 1×10^5^ cells were spun down at 500 g for 5 min., resuspended in HBSS^plus^ and transferred to 96 well U-bottom plates (Corning #7007) before staining/labeling. Samples were assayed on a BD Accuri C6 flow cytometer with 488 nm and 640 nm lasers and FL-1 533/30 nm, Fl-2 585/40 nm, FL-3 670LP and FL-4 (675/25 nm) photomultipliers. Per sample, 1×10^5^ events were collected and analyzed using BD Cflow analysis software.

For cell cycle analysis, cells fixed by drop-wise addition of −20°C absolute EtOH to a final concentration of ∼70% were collected at 750 g, resuspended in 3.8 mM Na-citrate, pH 7 (Sigma, #71498) and stained by addition of 50 µg/ml propidium iodide (Life Techn. P-3566, Ex./Em. 535 nm/617 nm), 10 µg/ml RNase A (Roche #11119915001) and 0.05% TX-100 (SIGMA #T8787) for 60 min at RT before analysis.

For determination of metabolic activity, membrane asymmetry and membrane permeability, NPCs were assayed with C_12_-Resazurin [37] (Vega-Avila and Pugsley, 2011), APC-Annexin-V [38] and SYTOX Green [39] (Life Techn. #V35114). NPCs were washed in Annexin binding buffer (ABB: 100 mM HEPES, 140 mM NaCl, 25 mM CaCl2, pH 7.4) and resuspended in 100 µl ABB containing 500 nM C_12_-Resazurin, 5 ul Alexa-Fluor 647 Annexin-V conjugated antibody (Ex./Em. 650/665 nm, Life Techn. #A23204) and 10 nM SYTOX Green. After incubation for 15 min at 37°C, cells were washed twice with ABB and assayed at 530 nm (SYTOX Green, FL-1), 575 nm (C_12_-Resazurin, Fl-2) and 660 nm (APC Annexin-V, FL-3).

### ATP production

2×10^4^ cells/mm^2^ cells seeded in 96-well plates 48 hrs before the experiment were treated as described under “NPC Treatment”, then lysed on ice and assayed with a coupled luciferin/luciferase assay (ATPLite, Perkin Elmer, #6016941) according to the manufacturer’s instructions. Plates were read on a VERITAS Luminometer (Turner Biosystems #998-9100). ATP content as determined by an ATP standard was then normalized to protein content determined by BCA Assay.

### Organelle-specific protein import

Adherent NPC cultures seeded in chamber slides and grown to 100% confluence were transduced with two baculoviral vectors (Cell Light, BacMam 2.0 system, Life Techn.) targeted to either the peroxisomal (CellLight Peroxisome-GFP, #C10604) or the mitochondrial (CellLight Mitochondria-RFP, #C10601) compartment according to the manufacturer’s instructions at M.O.I.s of 50. Development of organelle-specific fluorescent protein expression was evaluated in live cells by fluorescence microscopy.

### Proteasome Activity

Protein aggregation in NPCs was assayed in adherent NPCs cultured with 20 µM rotenone alone or with 10 µM of the proteasome inhibitor MG132 (ProteoStat aggresome detection Kit, Enzo Life Sciences #ENZ-51035-K100). NPCs, either on slides (for fluorescence microscopy) or detached from substrate (for flow cytometry) were fixed and permeabilized and then stained with 5 µM of the aggresome/proteasome specific dye Bodipy TMR-AHX3L3VS (Ex./Em. 500/600 nm) and Hoechst 33342 according to the manufacturer’s instructions. From flow cytometry of NPCs, aggregesome propensity factors (APF) were calculated from the mean RFU (MRFU) of Bodipy-TMR fluorescence: APF = 100×[MRFU MG132 treated cells−MRFU untreated cells]/MRFU MG132 treated cells.

### Cytochrome c release

Subcellular fractionation of cultured NPCs was done as previously described [40]. All steps were performed at 4°C. 1×10^7^ cells treated with 4 µM PQ for 1 hr were harvested and resuspended in 500 µl of mitochondrial isolation buffer (250 mM sucrose, 1 mM EDTA, 1 mM DTT, 10 mM HEPES, pH 7.5) supplemented with protease inhibitor (Complete Ultra, Roche #11836153001) and 1 mM DNase I (Roche #03724778103). Cells were fractionated by 3 passages through a 30G needle, centrifuged at 1000 g for 10 min. The supernatant was then spun at 10.000 g for 15 min to collect cell organelles and the supernatant was concentrated with a 3 kD Microcon filtration unit (YM-3 Millipore #42420) at 14.000 g for 30 min. Protein concentration was determined by BCA-Assay and 30 µg protein were used for immunoblotting.

### Statistical analysis

For individual experiments the mean ± SD or mean ± SEM were plotted. Non-directional Student’s t test was used for direct statistical comparisons. For multiple comparisons, multivariant ANOVA was used. Where significant *F*-values were obtained, pair wise comparisons were made using Wilcoxon–Mann/Whitney *post hoc* analysis. Differences were considered statistically significant at *p*≤0.05.

## Results

### Normal morphology but reduced proliferative capacity in SNCA-Tri NPCs

**Figure 1:**
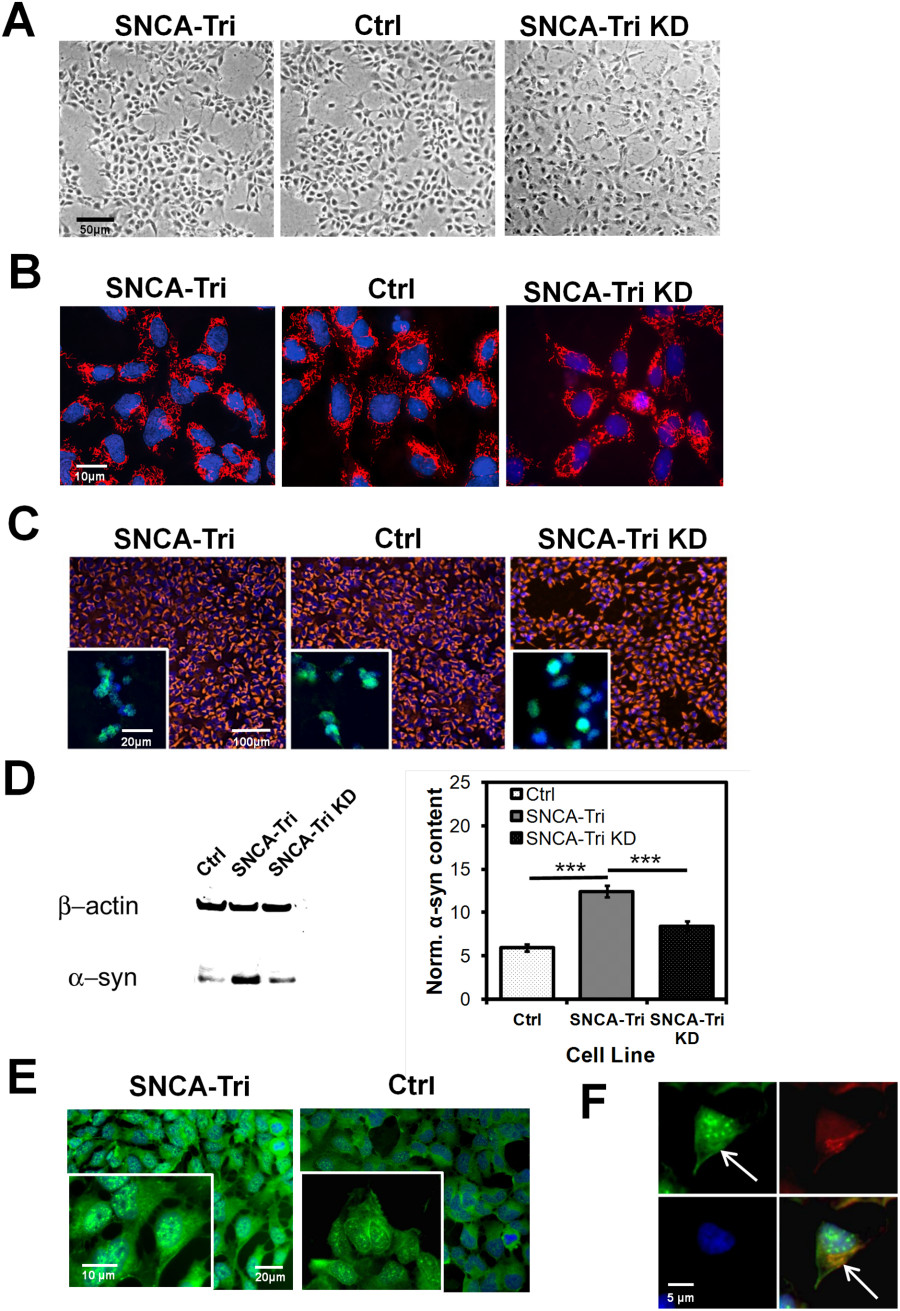
NPC characterization. **A) Phase contrast microscopy** of α-synuclein gene triplication (SNCA-Tri), control (Ctrl) and α-synuclein knockdown (SNCA-Tri KD) iPSC-derived NPC lines (Scale bar: 50 µm) shows normal cell morphology. **B)** **Mitochondrial and nuclear morphology** of NPCs visualized by fluorescence microscopy using Mitotracker Red CMX Ros (red) and Hoechst 33342 (blue) (Scale bar: 10 µm). **C)** **Stem cell marker expression**. Immuno-cytochemistry on fixed NPCs detecting cytoplasmic Nestin expression pattern with secondary Alexa 588 conjugated antibody (orange) by fluorescence microscopy (Scale bar: 100 µm). Insert: Immuno-cytochemistry for the nuclear stem cell marker SOX1, detected by a secondary Alexa-488 conjugated antibody (green) (Scale bar: 20 µm). Nuclear counter stain by Hoechst 33342 (blue). **D) Representative α-synuclein protein expression** patterns (left) by immunoblot of protein lysates from a control line (Ctrl), the SNCA-Tri NPC line and the corresponding α-synuclein knock down line (SNCA-Tri KD) with β-actin serving as loading control. Right: Quantification of β-actin normalized α-synuclein expression levels (n = 4, mean ± SEM, Ctrl/SNCA-Tri/SNCA-Tri KD: 12.4/5.9/8.3, ***p≤0.001, t-test; from two independent experiments). **E) ICC of α-synuclein protein expression in adherent NPCs** detected by a polyclonal α-syn antibody and visualized by Alexa-488 conjugated secondary antibody (green). DAPI nuclear counterstain (blue); (Scale bar 20 µm). Insert: Higher magnification image (Scale bar: 10 µm). **F)** **Colocalization of subcellular** **α-synuclein distribution with mitochondria** in adherent NPCs labeled with Mitotracker Red CMX Ros (red) and probed for α-syn as under E) (Scale bar: 5 µm).

Patient-derived NPC lines carrying the *SNCA* gene triplication (SNCA-Tri), the SNCA-Tri shRNA knockdown line (SNCA-Tri KD) and those from two unaffected controls (Ctrl) were morphologically indistinguishable under normal, high glucose growth conditions (HG) (**Figure 1A**) and did not show differences in mitochondrial shape or content (**Figure 1B**). All three NPC lines expressed neuronal stem cell markers Nestin and SOX1 (**Figure 1C**). Confluent cultures of SNCA-Tri NPCs expressed twice as much α-syn compared to controls irrespective of passage number, and SNCA shRNA knockdown resulted in a significant reduction of SNCA expression levels in NPCs (n = 4, mean ± SEM, SNCA-Tri/Ctrl: 12.4/5.9, SNCA-Tri KD: 8.3, ***p≤0.001, t-test) (**Figure 1D**). The differential α-syn expression levels in NPCs were also observed by ICC, with the protein associated with sub-cellular structures such as with mitochondria (**Figure 1E** **/F**).

**Figure 2:**
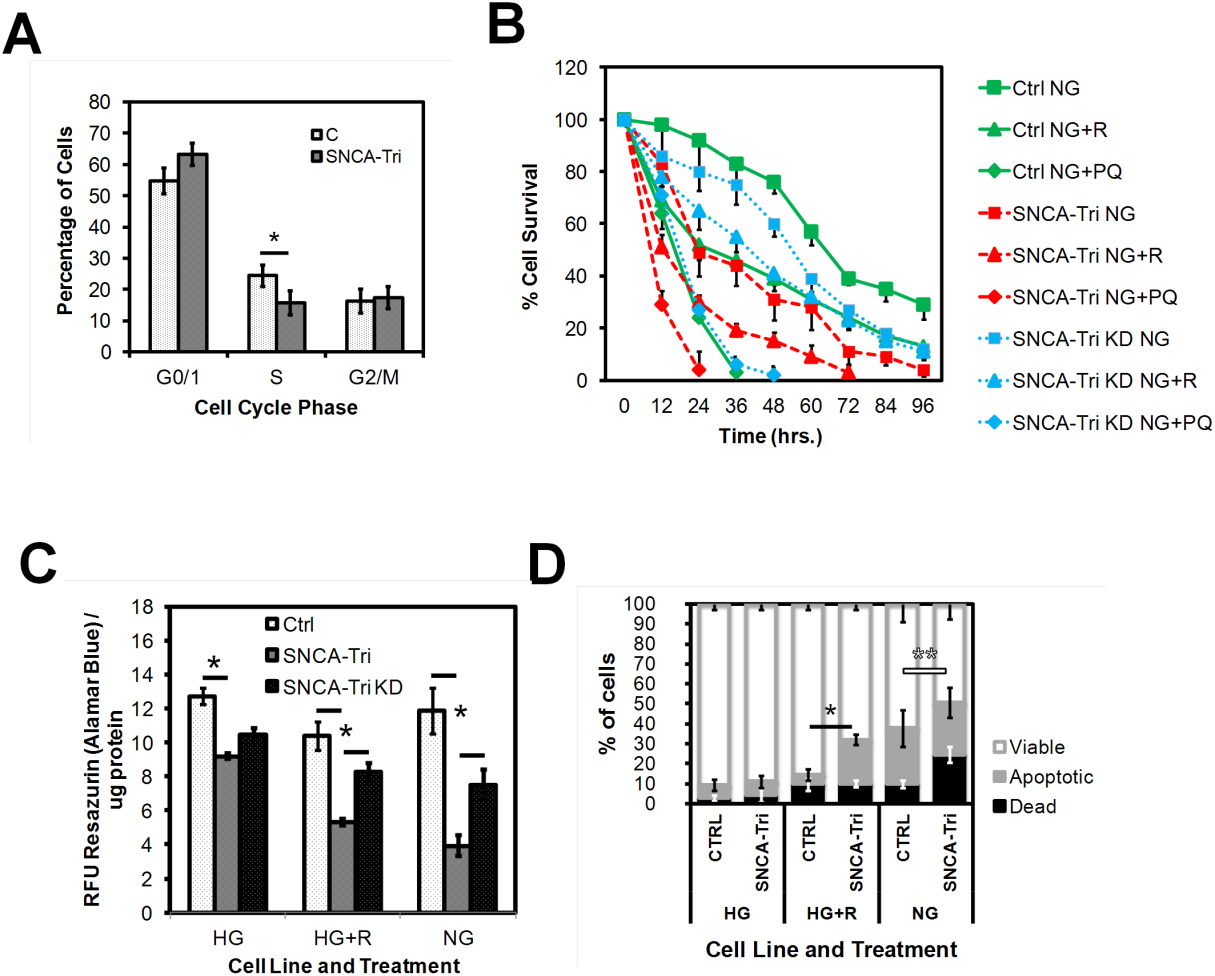
NPC viability. **A) Cell cycle analysis** by propidium-iodine (PI) staining and flow cytometry analysis of Ctrl and SNCA-Tri NPCs with staining grouped by cell cycle phase (G0/1, S and G2/M), showing a reduced percentage of SNCA-Tri NPCs in the S phase (n = 3, mean ± SD, *p = 0.047). **B)** **Survival under nutritional and toxicant stress.** NPCs propagated in medium without glucose (NG) untreated or treated with 20 µM rotenone (R) or 20 µM paraquat (PQ). Survival curves (every 12 hours) for the Ctrl, SNCA-Tri and SNCA-Tri KD cell lines after analysis of adherent cell count (ImageJ). Percentage of surviving cells with time (hrs) (n = 3, mean ± SEM). **C)** **Cell viability assayed by plate reader based high throughput screen (HTS)** of NPCs untreated (HG), treated with 20 µM rotenone (HG+R) or without glucose (NG) for 18 hrs. Live cells were stained with 1 µM of the RedOx indicator C_12_-Resazurin/Alamar Blue for 15 min before analysis. Graphed are endpoint fluorescence units (RFU) normalized to total cellular protein/well (ug protein) (n = 3, mean ± SEM, *p≤0.05). **D)** **Cell viability assayed by** flow cytometry evaluation of apoptosis and cell death in live NPCs treated as under A). Cells stained with C_12_-Resazurin for cell viability and with Sytox-Green. Graphed are percentages of metabolic active NPCs, determined by Resarufin (Ex./Em. 563/587 nm) fluorescence (viable), apoptotic cells (cell membrane asymmetry detected by an Annexin-V Alexa-660 nm conjugated antibody) (n = 3, mean ± SD, Ctrl/SNCA-Tri: 5.3%/24.4%, *p = 0.027) or cell death (nuclear fragmentation, detected by Sytox-Green, Ex./Em. 488/530 nm) (n = 3, mean ± SD, Ctrl/SNCA-Tri: 5.3%/24.4%, **p = 0.004).

Changes in cell cycle have been observed in other progenitor cell types used in models for neurodegeneration [41]. Under naïve conditions, cell cycle analysis showed a reduction in S-Phase DNA content of SNCA-Tri NPCs (n = 3, mean ± SD, 24.4%/15.7%, *p = 0.047, t test), suggestive of a delay in G1/S–phase transition at the G1 restriction point and resulting in reduced proliferative capacity (**Figure 2A**).

To investigate the impact of starvation and toxicants on SNCA-Tri NPC viability and stress resistance, we established protocols to imitate general metabolic and specifically mitochondrial stress conditions. We developed a starvation protocol that omitted glucose from the culture medium and a toxicant stressor panel that exposed NPCs to environmental toxins and toxicants such as rotenone (R), paraquat (PQ), staurosporine (SP) and oligomycin (O).

SNCA-Tri NPCs grown without glucose supplementation (NG) and exposed to 20 µM Rot or 20 µM PQ showed progressive cell death over the course of 4 days (% of surviving cells), that was attenuated in the SNCA-Tri KD NPCs (**Figure 2B**) (n = 3 from 2 clones for Ctrl and SNCA-Tri, mean ± SEM). When analyzing viability of these cell lines by HTS using the fluorescent RedOx indicator Resazurin/Resarufin, SNCA-Tri clones showed significantly reduced viability under all treatment conditions (n = 3, mean ± SEM, HG: 12.7/9.2, HG+R: 10.4/5.3, NG: 11.8/4.0, *p≤0.05). Knockdown of α-syn in the SNCA-Tri KD NPCs, resulted in significantly improved viability under stress conditions (SNCA-Tri/SNCA-Tri KD: HG+R: 5.3/8.3, NG: 4.0/7.5) (**Figure 2C**). These results were confirmed by flow cytometry analysis of Ctrl and SNCA-Tri NPCs, simultaneously assaying the number of viable, apoptotic (membrane inversion detected by Annexin-V) and dead (Sytox positive) NPCs. A significant higher percentage of SNCA-Tri NPCs displayed apoptotic behavior under rotenone stress (n = 3, mean ± SD, Ctrl/SNCA-Tri: 5.3%/24.4%, *p = 0.027). Under glucose starvation (NG) conditions, significantly increased cell death was observed in SNCA-Tri NPCs (n = 3, mean ± SD, Ctrl/SNCA-Tri: 5.3%/24.4%, **p = 0.004) (**Figure 2D**).

### Altered cellular energy balance and decreased mitochondrial function in SNCA-Tri NPCs

**Figure 3:**
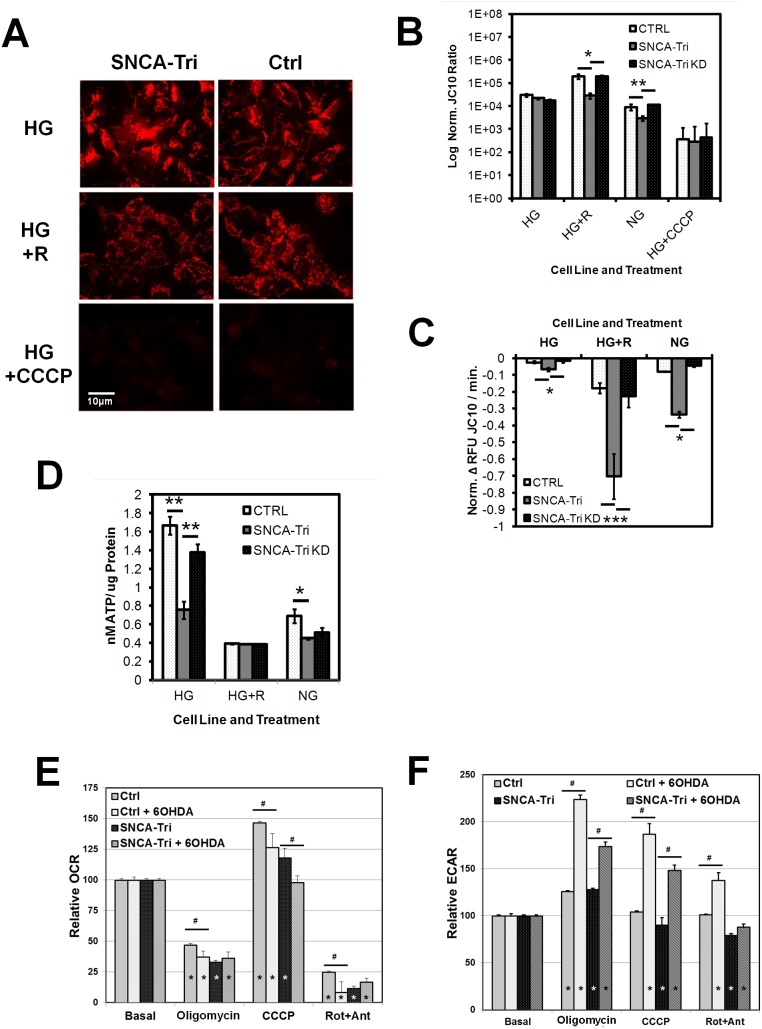
Mitochondrial membrane potential (MMP) and energy balance. **A) Fluorescence microscopy** of MMP in live NPCs from patient (SNCA-Tri) and control (Ctrl) loaded with 100 nM TMRM in normal growth medium (HG), medium plus 20 µM Rotenone (HG+R) or with 1 µM of the ionophore CCCP (HG+CCCP) as negative control (Scale bar: 10 µm). **B)** **Plate reader based high throughput screen (HTS) of MMP** in live NPCs loaded with 20 µM JC-10 for 45 min. Cells were also treated with medium w/o glucose (NG). Shown are log ratios of reduced (Ex./Em. 540 nm/590 nm) to oxidized JC-10 (Ex./Em. 488 nm/520 nm) normalized to Hoechst 33342 (Log Norm. JC-10 Ratio) after 60 min. (n = 8, mean ± SEM, Ctrl/SNCA-Tri/SNCA-Tri KD for HG+R: 202/29/194 (xE04), *p≤0.05; for NG: 92/30/118 (xE03) **p≤0.006). **C) Plate reader based HTS for MMP loss** in live NPCs prepared and analyzed as under B). Fluorescence measurements were acquired as under B) every 5 min for 10 cycles and loss of MMP with time graphed as ΔRFU/min. (n = 8, mean ± SEM, Ctrl/SNCA-Tri/SNCA-Tri KD: HG: −0.02/−0.06/−0.01, *p≤0.05; HG+R: −0.17/−0.70/−0.22 ***p<0.001, NG: −0.08/−0.33/−0.04, *p≤0.05). **D)** **Luminescence plate reader based HTS** **of ATP levels** in Ctrl, SNCA-Tri and SNCA-Tri KD NPCs under the above growth conditions (HG, HG+R, NG) assayed by a coupled luciferin/luciferase assay. Depicted are ATP contents in cells treated with 20 µM rotenone (R) for 18 hrs. (n = 8, mean ± SD nMATP/ug protein in: Ctrl/SNCA-Tri/SNCA-Tri KD: HG: 1.66/0.75/1.37, **p = 0.003; NG: 0.69/0.45/0.51, *p = 0.04). **E and F) Mitochondrial metabolic activity** studied by Seahorse XF24 analysis. **E)** Oxygen Consumption Rate (OCR) and **F)** Extracellular Acidification Rate (ECAR). Shown are relative OCR compared to basal values as a function of the sequential addition of mitochondrial inhibitors Oligomycin (1 µM), CCCP (1.5 µM) and Rotenone (Rot, 5 µM) + Antimycin A (Ant, 1 µM). Significant changes compared to basal OCR rates (*p<0.05) and differences between lines treated with and without 6-OHDA (250 µM) for 1 hr are indicated by # (#p<0.05, mean ± SEM, n≥17; from five independent experiments).

We next investigated energy status and metabolism in SNCA-Tri NPCs. When SNCA-Tri NPCs challenged with rotenone and loaded with tetramethylrhodamine (TMRM) were examined by fluorescence microscopy, they showed reduced mitochondrial membrane potential (MMP) (**Figure 3A**). We then quantified MMP by HTS fluorescence plate reader analysis using the ratiometric fluorescent dye JC-10. MMP in SNCA-Tri NPCs was significantly more impaired by rotenone and glucose withdrawal compared to controls, and knockdown of α-syn reinstated normal MMP levels in SNCA-Tri KD NPCS (n = 8, mean ± SEM, Ctrl/SNCA-Tri/SNCA-Tri KD for HG+R: 202/29/194 (x10^4^), *p≤0.05; for NG: 92/30/118 (x10^3^) **p≤0.006) (**Figure 3B**). In addition, when monitoring MMP over time, SNCA-Tri NPCs lost their MMP significantly faster than Ctrl NPCs when exposed to stress by extended HTS analysis (1 hr), which was compounded by rotenone stress and glucose withdrawal and could be returned to rates in Ctrl by knockdown of α-syn (n = 8, mean ± SEM, Ctrl/SNCA-Tri/SNCA-Tri KD: HG: −0.02/−0.06/−0.01, *p≤0.05; HG+R: −0.17/−0.07/−0.22 ***p<0.001, NG: −0.08/−0.33/−0.04, *p≤0.05) (**Figure 3C**).

Cellular ATP content in SNCA-Tri cells compared to controls was reduced both under normal growth conditions and under glucose starvation, indicating a metabolic deficit in SNCA-Tri NPCs that could be ameliorated by knockdown of α-syn in the SNCA-Tri KD NPCs (n = 8, mean ± SD nM ATP/ug protein in: Ctrl/SNCA-Tri/SNCA-Tri KD: HG: 1.66/0.75/1.37, **p = 0.003; NG: 0.69/0.45/0.51, *p = 0.04 (**Figure 3D**).

To investigate mitochondrial function in live NPCs we measured cell respiratory control in live NPCs (**Figure 3E**). SNCA-Tri NPCs showed altered O_2_ consumption rates and non- mitochondrial respiration (Ctrl/SNCA-Tri: 24%/8%), and OCR was sensitive to the neurotoxin 6-hydroxy-dopamine (6-OHDA). Relevant for mitochondrial function, spare respiratory capacity, representing the ability of mitochondria to respond to an increase in energy demand (expressed as the quantitative difference between basal OCR and maximal uncontrolled OCR) by addition of the uncoupler CCCP was reduced from 57% in control NPCs to 34% in SNCA-Tri NPCs (n≥17, mean ± SEM, (#p<0.05). When NPCs were exposed to the neurotoxin 6-OHDA their spare respiratory capacity was further decreased, with the difference between Ctrl and SNCA-Tri NPCs being preserved (37%/14%). Mitochondrial proton leak, represented by the reduction of OCR in presence of oligomycin and indicative of mitochondrial uncoupling from respiration, was significantly higher in SNCA-Tri NPCs (OCR Ctrl/SNCA-Tri: 48%/32%), supporting the observations of decreased coupling efficiency and less stable MMP in SNCA-Tri NPCs.

The glycolytic activity of both lines (measured by medium acidification and graphed as extracellular acidification rate (ECAR)) **(** **Figure 3F** **)** was similar in Ctrl and SNCA-Tri NPCs, as indicated by comparable levels of lactic acid formation after inhibition of mitochondrial ATP production, but ECARs in SNCA-Tri cells stressed by 6-OHDA were significantly decreased, confirming the findings from the plate reader based HTS screen for ATP levels.

### Delayed protein import and increased protein aggregation in SNCA-Tri NPCs

**Figure 4:**
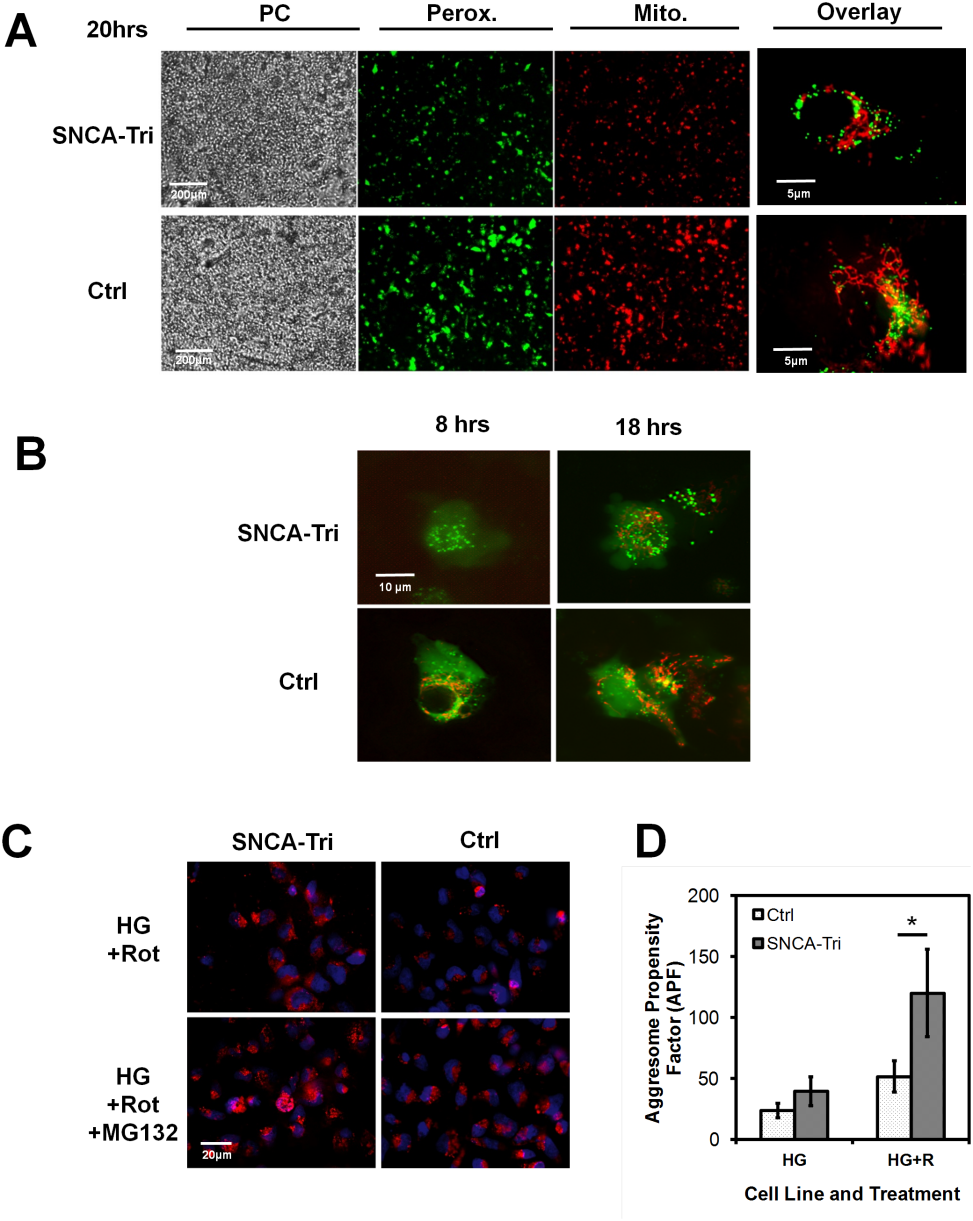
Protein biosynthesis and proteasome function. **A) Mitochondrial protein biosynthesis and protein import.** Fluorescent protein expression patterns in confluent adherent NPC cultures (PC: Phase Contrast) transduced with two baculoviral vectors expressing fluorescent proteins targeted to either the peroxisomal (Perox.; Green) or the mitochondrial (Mito.; Red) compartment. Shown are fluorescent protein expression patterns in live confluent Ctrl and SNCA-Tri cell lines grown under normal growth conditions (HG) and evaluated 20 hrs post transduction (Scale bar: 200 µm, 5 µm). **B) Time resolved peroxisomal and mitochondrial protein biosynthesis**. Fluorescent protein expression patterns as under A), but imaged at 8 and 18 hrs post viral transduction. **C)** **Proteasome activity measured by fluorescence microscopy** of adherent NPCs cultured with 20 µM rotenone alone or with 10 µM of the proteasome inhibitor MG132. Depicted are fixed cells stained with 5 µM of the aggresome/proteasome specific dye Bodipy TMR-AHX3L3VS (red). Hoechst 33342 was used as nuclear counter stain (blue) (Scale bar: 20 µm). **D)** **Proteasome activity measured by flow cytometry** evaluation of cells treated and stained as under B). Charted are the aggresome propensity factors (APF) of NPCs calculated from the mean RFU (MRFU) of Bodipy-TMR fluorescence (APF = 100×[MRFU MG132 treated−MRFU untreated]/MRFU MG132 treated (n = 3, mean ± SD, APF Ctrl/SNCA-Tri: 51/120, *p = 0.041).

Protein biosynthesis and import into cellular organelles are essential for cellular anabolic metabolism [42]. When we transduced adherent NPCs with viral BacMam vectors encoding mitochondrial- and peroxisomal-targeted fluorescent proteins, we observed both delayed appearance and reduced levels of organelle-specific fluorescence in SNCA-Tri NPCs (**Figure 4A** **/B**).

On the catabolic side, dysfunction of the mechanisms to repair and remove abnormal proteins, such as impaired unfolded protein response and proteasome function, have been shown to play a pivotal role in PD disease progression [43], [44]. When we analyzed cytoplasmic protein aggregates (aggresomes) in proteasome-inhibitor treated SNCA-Tri and Ctrl NPCs by microscopy and semi-quantitative flow cytometry (**Figure 4C, D**), we observed a significant increase in aggresome formation in rotenone and proteasome inhibitor-treated SNCA-Tri cells ( = 3, mean ± SD, APF Ctrl/SNCA-Tri: 51/120, *p = 0.041), suggesting increased activity of the cellular proteasome system.

### Increased cellular stress and reactive oxygen species (ROS) in SNCA-Tri NPCs

**Figure 5:**
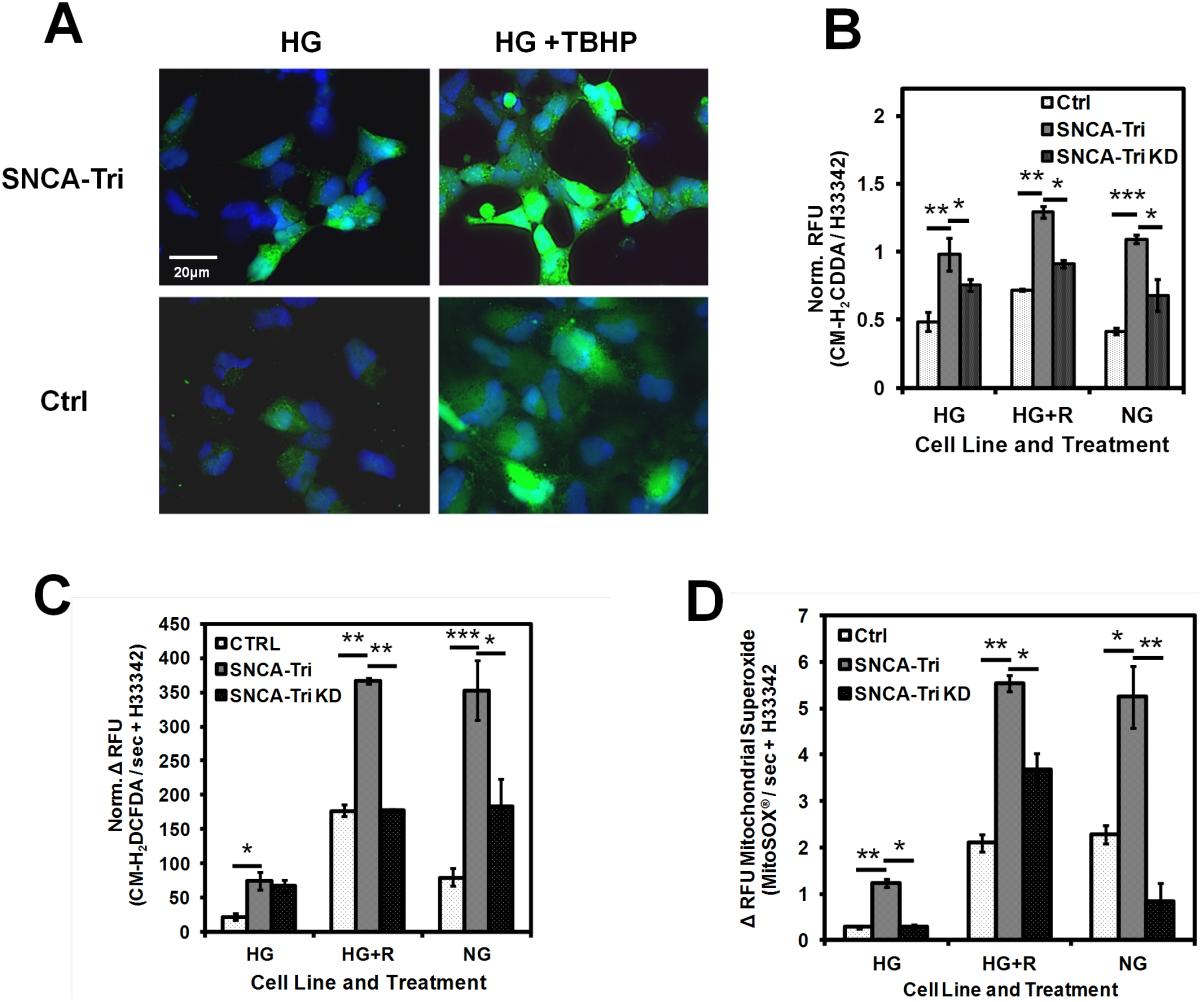
Reactive oxygen species (ROS) production. **A) Fluorescence microscopy** of live adherent NPCs untreated (HG) or treated with 100 µM TBHP (HG+TBHP), loaded with CM-H_2_DCFDA and imaged under controlled exposure conditions (10 sec fluorescent light exposure before image acquisition). Hoechst 33342 was used as counter stain (Scale bar: 20 µm). **B) Plate reader based HTS** of ROS levels in adherent NPC in 96-well plates and treated as under A). Relative CM-H_2_DCFDA fluorescence intensities (RFU) were normalized to Hoechst 33342 (H33342) (n = 12, mean ± SEM, Ctrl/SNCA-Tri/SNCA-Tri KD: HG: 0.5/1/0.75, HG+R: 0.7/1.3/0.6, NG: 0.4/1.1/0.7, *p≤0.046, **p≤0.009, ***≤0.001). **C) ROS production rates** by HTS plate reader analysis of CM-H_2_DCFDA fluorescence development over time (Δ RFU CM-H_2_DCFDA/sec + H33342) in cells exposed to TBHP as under A), measured with normal medium (HG) with or without rotenone (R) and in medium without glucose (NG) (n = 12, mean ± SEM, Ctrl/SNCA-Tri/SNCA-Tri KD: HG: 22/75/68, HG+R: 177/367/178, NG: 80/353/184, *p≤0.010, **p≤0.007, ***p≤0.001). **D) Mitochondrial superoxide production** **rates** assayed by HTS plate reader analysis of the mitochondrial targeted fluorescent superoxide indicator MitoSOX. Depicted are changes in relative fluorescence units normalized to H33342) (Δ RFU MitoSOX/min + H33342) (n = 4, mean ± SD, Ctrl/SNCA-Tri/SNCA-Tri KD: HG: 0.28/1.2/0.3, HG+R: 2.1/5.5/3.7, NG: 2.3/5.2/0.8,*p≤0.038, **p≤0.007).

ROS have been shown to play an important role in PD disease progression [24], [25], [45]. By fluorescence microscopy, SNCA-Tri NPCs loaded with ROS indicator CM-H_2_DCFDA showed elevated basal ROS levels that were more prominent when cells were treated with the ROS generator tert-butyl-hydroxy-peroxide (TBHP) (**Figure 5A**). We confirmed the increased ROS burden on SNCA-Tri NPCs by semi-quantitative HTS analysis of ROS steady state levels (**Figure 5B**) and the rate of ROS generation in adherent NPCs (**Figure 5C**). Both the basal ROS levels (n = 12, mean ± SEM, Ctrl/SNCA-Tri/: HG: 0.5/1 **p = 0.002, HG+R: 0.7/1.3 **p = 0.046, NG: 0.4/1.1 ***p≤0.001) and ROS production rates (n = 12, mean ± SEM, Ctrl/SNCA-Tri HG: 22/75 *p = 0.010, HG+R: 177/367 **p = 0.002, NG: 80/353 ***p≤0.001) in SNCA-Tri NPCs were significant elevated when compared to Ctrl. When α-syn expression was knocked down, NPCs displayed significantly reduced ROS steady state (n = 12, mean ± SEM, SNCA-Tri/SNCA-Tri KD: HG: 1.0/0.75 *p = 0.002, HG+R: 1.3/0.6, * = 0.046) and ROS production rates (n = 12, mean ± SEM, SNCA-Tri/SNCA-Tri KD: HG+R: 367/178 **p = 0.007, NG: 353/184, *p = 0.015).

Increased cellular superoxide production has also been implicated in PD pathology [46]. HTS analysis of NPCs labeled with a mitochondria-specific fluorescent superoxide indicator showed increased superoxide levels in SNCA-Tri NPCs that was compounded by exposure to cellular stress and was reduced by knockdown of α-syn (n = 4, mean ± SD, Ctrl/SNCA-Tri/SNCA-Tri KD: HG: 0.28/1.2/0.3, HG+R: 2.1/5.5/3.7, NG: 2.3/5.2/0.8, *p≤0.038, **p≤0.007) (**Figure 5D**). The higher mitochondrial superoxide levels were also confirmed by flow cytometry analysis using the same exposure conditions (data not shown). These results suggest defects in scavenging and clearing cellular ROS and/or mitochondrial superoxide levels in SNCA-Tri NPCs, or may indicate a more complex regulation and balance of ROS and superoxide levels in neural progenitor cells [47].

### Impaired mitochondrial integrity and permeability transition in SNCA-Tri NPCs

**Figure 6:**
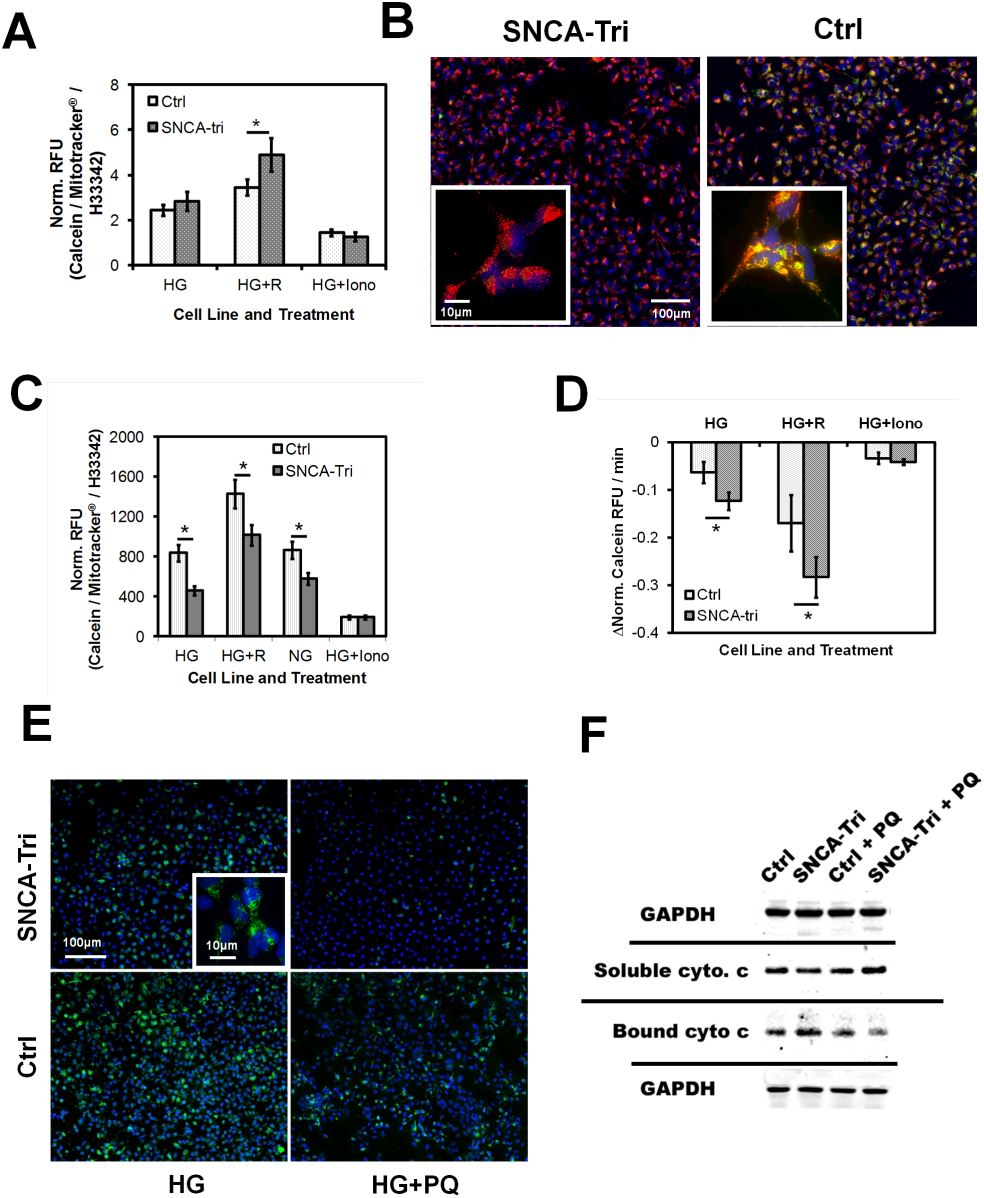
Mitochondrial integrity, MPT opening, and apoptosis. **A) Mitochondrial calcein loading** by fluorescent plate reader HTS of in NPCs grown in 96 well micro plates. Relative fluorescent signal intensities (RFU) for calcein acquired after 30 min loading with Calcein AM and CoCl_2_ were normalized to mitochondrial content (Mitotracker) and to cell number by Hoechst 33342 (H33342). 1 µM ionomycin was added directly before HTS analysis as negative control (Iono) (n = 8, mean ± SD, Ctrl/SNCA-Tri: 3.4/4.9, *p = 0.039). **B)** **MPT-induced mitochondrial calcein loss** in Ctrl and SNCA-Tri NPCs after mitochondrial calcein–AM loading. Representative fluorescence microscopy images of Ctrl and SNCA-Tri NPCs loaded with calcein (green), Mitotracker (red) and CoCl_2_ were assayed 1 hr. after treatment with 4 µM staurosporine under NG conditions. MPT opening results in entry of CoCl_2_ into mitochondria and loss of calcein signal (nuclear counter stain: Hoechst 33342; scale bar: 100 µm). **Inserts:** Higher magnification images obtained by conventional fluorescence microscopy (Scale bar: 10 µm). **C) HCI automated fluorescence microscopy analysis** of MPT in NPCs treated with 4 µM staurosporine as under B). Images (see B) were analyzed using MetaXpress image processing software. Depicted are data of cellular calcein signal intensities normalized to mitochondrial content (Norm. RFU Calcein/RFU Mitotracker) from two replicate wells with four image sites/well per treatment condition (n = 16, mean ± SD, Ctrl/SNCA-Tri, HG: 834/457, HG+R: 1425/1011, NG: 864/574, HG+Iono: 187/190, *p≤0.01). **D)** **Kinetic evaluation of MPT opening** and loss of mitochondrial calcein signal after induction of MTP using fluorescence plate reader based HTS analysis. NPCs treated and prepared as under B) were loaded with 4 µM stauropsporine and changes in calcein signal normalized to cell number and mitochondrial content (Δ Norm. RFU) were recorded every 1 min for 20 min (n = 8, mean ± SD, Ctrl/SNCA-Tri, HG: −0.06/−0.12, HG+R: −0.17/−0.28, HG+Iono: −0.03/−0.04, *p≤0.01). **E)** **Cytochrome c immuno-cytochemistry** in Ctrl and SNCA-tri NPCs challenged with 200 µM paraquat (PQ) 15 min. before fixation. Shown are permeabilized cells probed with cytochrome c antibody, detected by an Alexa-488 nm labeled secondary antibody (green). Cells were counter stained with Hoechst 33342 (blue) (Scale bar: 100 µm, insert: 10 µm). **F)** **Immunoblot analysis of cytochrome c levels** in sub-cellular fractions containing either cellular organelles (containing bound cytochrome c) or cytosolic proteins (with soluble cytochrome c) from NPC cell lysates (Ctrl and SNCA-Tri) treated with paraquat (PQ) as under E). Cytochrome c (14 kDa) and GAPDH (40 kDa) specific antibodies were detected by a secondary IR-dye conjugate.

Opening of the mitochondrial permeability transition pore (MPT) results in mitochondrial depolarization, uncoupling of oxidative phosphorylation, large-amplitude mitochondrial swelling and ultimately apoptosis [23]. We monitored mitochondrial integrity and MPT opening in live NPCs by loading mitochondria with fluorescent calcein in presence of a cytoplasmic calcein quencher and conducted both endpoint analysis of mitochondrial calcein levels and kinetic studies on the rate of mitochondrial calcein loss. HCI analysis revealed higher mitochondrial calcein loading capacity in SNCA-Tri NPCs challenged with rotenone (n = 8, mean ± SD, Ctrl/SNCA-Tri: 3.4/4.9, *p = 0.039), indicative of increased cellular and mitochondrial stress (**Figure 6A** **)**. To investigate the mitochondrial resilience to toxin-induced MPT opening, we analyzed mitochondrial calcein fluorescence in NPCs after treatment with 4 µM staurosporine by HCI. The SNCA-Tri NPC populations showed a significantly reduced mitochondrial calcein signal, indicative of more pronounced MPT opening compared to Ctrl NPCs (n = 3, mean ± SD, Ctrl/SNCA-Tri, HG: 834/457, HG+R: 1425/1011, NG: 864/574, HG+Iono: 187/190, *p≤0.01) under all experimental conditions (**Figure 6B, C**).

HCI analysis of the rate of mitochondrial calcein loss revealed a significantly faster loss of calcein signal in SNCA-Tri cells regardless of treatment regimen (n = 8, mean ± SD, Ctrl/SNCA-Tri, HG: −0.06/−0.12, HG+R: −0.17/−0.28, HG+Iono: −0.03/−0.04, *p≤0.01) that was concurrent with reduction in TMRM signal (data not shown) (**Figure 6D**). Taken together, these observations indicate both increased MPT opening and a faster rate of mitochondrial permeabilization in SNCA-Tri NPCs.

To confirm the loss of mitochondrial outer membrane integrity in toxicant exposed SNCA-Tri cells, we challenged NPCs with PQ to induce acute cell death and then performed cytochrome c immunocytochemistry. SNCA-Tri cells on average showed significantly reduced cytochrome c signal, confirming outer mitochondrial membrane permeabilization in these cells (**Figure 6E**). The increased mitochondrial cytochrome c loss in SNCA-Tri NPCs under these conditions was also confirmed by immunoblot analysis of cytochrome c content in cytosol- and organelle-enriched sub-cellular fractions from PQ treated NPCs. (**Figure 6F**).

### Caspase activation and apoptosis

As we had observed significant deficiencies in cellular catabolic and anabolic processes, energy metabolism and altered ROS levels as well as increased membrane asymmetry and cell death in challenged SNCA-Tri NPCs (see Figure 2D), we investigated the mechanism of NPC cell death.

**Figure 7:**
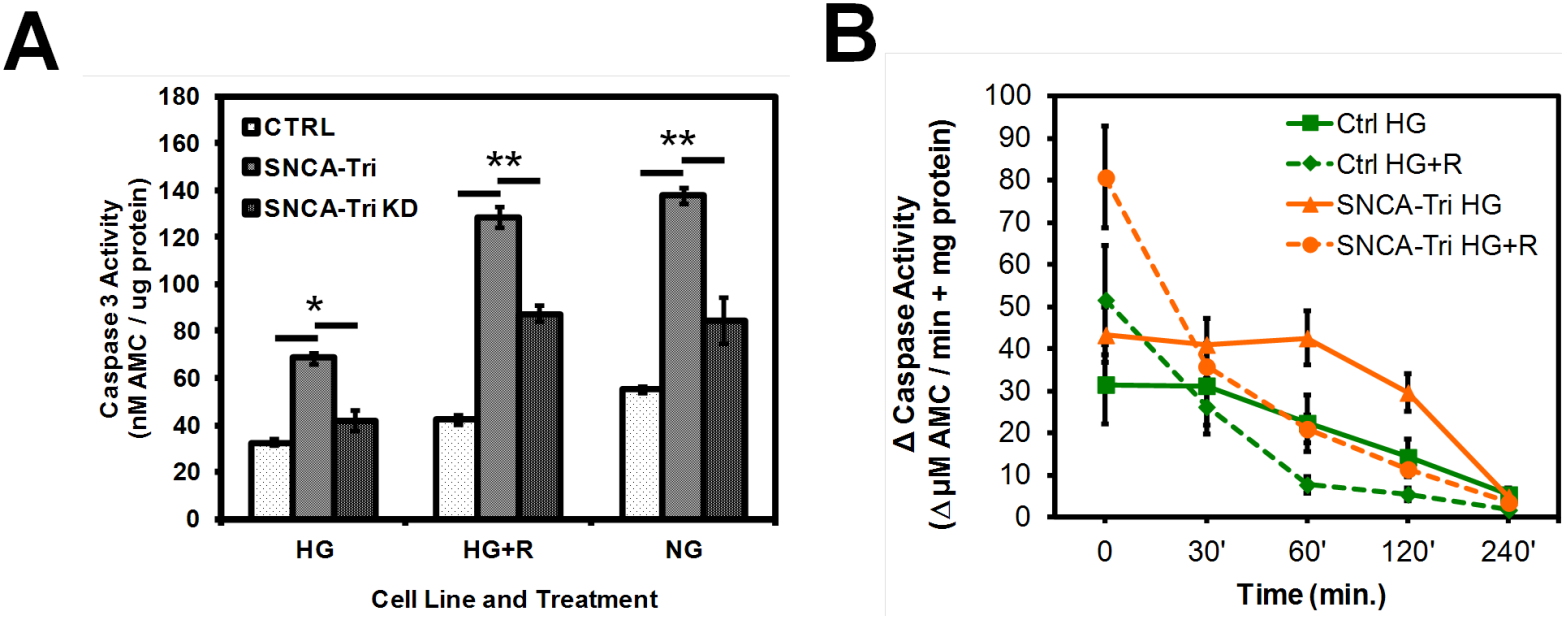
Apoptosis sensitivity and caspase activation. **A) Caspase 3 activity** in cell lysates from adherent NPCs either left untreated or treated with 20 µM rotenone (R) for 18 hrs and then exposed to 1 uM staurosporine for 120 min before analysis. HTS analysis for caspase 3 activity from cell lysates was by activation of the fluorescent caspase substrate 7-amino-4-methylcoumarin (AMC) (Ex./Em. 340/440 nm) (n = 9, mean ± SEM, Ctrl/SNCA-Tri/SNCA-Tri KD, HG: 33/69/42, HG+R: 42/129/87, NG: 55/138/85, *p≤0.050, **p≤0.0035; from three independent experiments). **B)** **Kinetics of caspase 3/7** **activity** in permeabilized NPCs pretreated as described under B) and assayed 15 min after staurosporine treatment. Changes in caspase 3 activity are depicted as ΔµM AMC fluorescence/min + mg cellular protein (detected by Bradford protein assay) (n = 9, mean ± SEM).

To determine the activity of effector caspase 3 in nutrient and toxicant stressed NPCs we performed HTS studies using a fluorescent caspase substrate. When comparing ratios of caspase activation between Ctrl and SNCA-Tri NPCs, α-syn overexpression resulted in a significant increase in caspase activation under all treatment conditions (**Figure 7A**) (n = 9, mean ± SEM, Ctrl/SNCA-Tri HG: 33/69 *p = 0.028, HG+R: 42/129 **p≤0.0085, NG: 55/138, **p = 0.0015). Knockdown of α-syn in the SNCA-Tri NPCs resulted in significantly reduced activation of capase 3, but activity did not return completely to the levels observed in controls (n = 9, mean ± SEM, SNCA-Tri/SNCA-Tri KD, HG: 69/42 *p = 0.050, HG+R: 129/87 **p = 0.0033, NG: 138/85 **p≤0.0023).

To better resolve temporal caspase activation in NPCs, we examined time resolved caspase 3 cleavage activity in permeabilized NPCs after 15 min exposure to staurosporine (**Figure 7B**). Under normal growth conditions (HG), both NPC lines showed stable caspase 3 activation during the first 60 min., with the SNCA-tri NPCs displaying higher levels of activation during this time. Under rotenone stress, both cell lines, but particular the SNCA-Tri NPCs displayed high caspase activation levels that decreased more rapidly, suggesting accelerated loss of cellular function compared to control and confirming the observations about faster loss of viability in SNCA-Tri NPCs.

## Discussion

The insight into molecular pathomechanisms responsible for neurodegeneration of Mendelian forms of parkinsonism is advancing rapidly, however little is known about the impact of these genetic defects on function and fate of human stem or neuronal precursor cells and their predisposition to nutritional and environmental stress. A few reports showed an impairment of embryonic neurogenesis in animal models for PD [48], [49]. and in a comprehensive screen in iPSC-derived neurons carrying mutations in PD genes has shown convergence of cellular disease mechanisms, such as increased cellular stress and mitochondrial dysfunction in these neurons [25]. However, the impact of these mutations on function, viability, and proliferative potential of human NPCs has not yet been investigated.

In this study, we have used NPCs derived from iPSCs carrying a triplication of the SNCA gene locus [10], [11], [24] to investigate the impact of this gene defect on neuronal precursor cell biology and specifically mitochondrial function and bioenergetics. To link the SNCA gene multiplication to the observed phenotype, we also generated a stable SNCA shRNA knock down NPC line to correct the α-syn overexpression (SNCA-Tri KD).

SNCA-Tri and Ctrl NPCs as well as the engineered knockdown line SNCA-Tri KD presented with normal cell morphology and stem cell marker signatures. Also, SNCA-Tri NPCs expressed twice the amount of α-syn protein, as expected from quantitative RT-PCR analysis (data not shown), which was reduced to near normal levels in the knock down cells.

### SNCA-Tri NPCs show reduced viability and metabolic capacity

To model the impact of nutritional and environmental challenges on the cellular networks and particularly mitochondria, we subjected NPCs to metabolic stress by exposure to the mitochondrial toxicant rotenone, free radical generators such as paraquat, or nutrient (glucose) deprivation. We observed slower proliferative capacity, reduced viability and decreased cell survival rates in SNCA-Tri NPCs under cellular stress conditions, which was alleviated by SNCA shRNA knock down in these NPCs, suggesting a direct impact of α-syn overexpression on cell cycle progression, cell survival, metabolic fitness and stress resistance.

Cellular stress significantly affected cellular ATP content and mitochondrial membrane potential (MMP) in SNCA-Tri NPCs, suggesting a reduced capacity of these NPCs to mitigate metabolic challenges and maintain mitochondrial functionality.

Metabolism in highly proliferative cells such as NPCs is tightly regulated, with strong control of cytoplasmic glycolysis and mitochondrial OXPHOS [50]. The observed changes in cellular ATP content in SNCA-Tri NPCs together with mitochondrial functional abnormalities point to disturbed coordination of cellular bioenergetics in these stem cells.

Metabolic flux analysis, measuring oxygen consumption rate and extracellular acidification, confirmed the mitochondrial membrane instability. The observed media acidification suggest changes in glycolytic flux in these NPCs, possibly leading to an imbalance of pyruvate and lactate and resulting in metabolic acidosis [51].

Taken together, these results point to de-synchronization of glycolytic and mitochondrial energy metabolism, resulting in reduced viability and proliferative capacity, supporting our previous findings in skin fibroblast cultures of this patient (Mak et al., 2011).

Respiratory chain function has consistently been found impaired in PD and in association with α-syn pathophysiology [15], and the observed association of α-syn with subcellular lipid bilayers suggests a general effect of overexpression on mitochondrial protein import and possibly respiratory chain functionality. This hypothesis is supported by the delayed appearance of transgenic fluorescent protein in SNCA-Tri NPC mitochondria, suggestive of altered mitochondrial protein import and assembly that could be a contributing factor to mitochondrial respiratory chain dysfunction.

### SNCA-Tri NPCs display increased cellular stress and ROS susceptibility

The ubiquitin proteasome system (UPS) is recycling dysfunctional cellular proteins [52], and impairment of this system plays an important role in neurodegenerative processes associated with PD [43]. Increased aggresome formation in toxicant-treated SNCA-Tri NPCs suggests that the UPS and proteasome activity may be heavily used, thus taxing the cellular catabolic systems. These data are also supported by the strong activity of peroxisomal matrix protein import observed in these NPCs.

Disturbed mitochondrial respiratory chain assembly and function result in increased ROS and superoxide production [53]. Similarly, altered protein transport and turnover result in increased oxidative stress in iPS-derived neuronal cultures [24], [26]. Increased ROS levels are consistently reported in PD pathophysiology [13], and we observed both higher steady state ROS levels and increased ROS production in SNCA-Tri NPCs; under normal growth conditions, under toxicant exposure, and nutrient deprivation. Our findings point towards increased ROS formation as consequence of the physiological changes observed in SNCA-Tri NPCs. In addition to the above direct ROS effects on mitochondrial function, the increased α-syn levels in SNCA-Tri NPCs could also impair intracellular ROS regulation by affecting mitochondrial anti-oxidant defense mechanisms, cellular RedOx balance and cell signaling [54].

Our data support a model where a-syn overexpression-initiated changes impair regulation and efficiency of cellular energy generation. These changes then lead to increased cellular stress, increased cellular ROS production and altered RedOx balance that could also affect NPC growth and differentiation.

### Increased mitochondrial apoptosis sensitivity of SNCA-Tri NPCs

Cellular stress is ascribed to induce transient or irreversible MTP opening, with the latter leading to initiation of mitochondria-mediated apoptosis. Under acute cellular stress, SNCA-Tri mitochondria showed hyperpolarization and increased mitochondrial calcein loading (indicative of increased calcium levels), which resulted in more rapid MTP opening. Mitochondria in connection with the endoplasmic reticulum are important components of cellular Ca^2+^ regulation [55], and dysregulation of this system has been shown to be part of the molecular pathomechanism in PD [56]. A-syn has been shown to increase mitochondrial Ca^2+^ uptake and impairs mitochondrial function [17], ultimately resulting in Ca^2+^ mediated mitochondrial apoptosis [57].

Mitochondrial toxicants such as rotenone raise mitochondrial ROS levels and increase mitochondrial Ca^2+^ stress [58]. In cell signaling, Ca^2+^ and Ca^2+^-dependent pathways regulate components of ROS/RedOx homeostasis [59], mitochondrial metabolic rate and ROS generation [60]. The higher cellular ROS burden, lower energy levels and higher propensity for MTP opening in connection with Ca^2+^ stress demonstrate the increased apoptosis sensitivity in SNCA-Tri NPCs [2]. As stem cells have a decreased capacity to mediate larger Ca^2+^ fluctuations and require tight control of ROS/RedOx during the onset of differentiation [61], abnormal Ca^2+^ homeostasis may also impact proliferation and differentiation of SNCA-Tri stem cells [62].

### Caspase Activation

Caspases have an important role both in the initiation of apoptotic events [63], [64], but also in sensitizing and priming cells to developmental changes [65]. When cultured under starvation conditions, SNCA-Tri NPCs displayed greater loss of viability and cell membrane integrity in response to mitochondrial toxicant treatment. Investigation of caspase activation patterns in toxicant and ROS challenged NPCs, showed significantly higher caspase activation in SNCA-Tri NPCs with functionally impaired mitochondria. In this context, our results also demonstrate the mitochondrial control of apoptosis initiation in NPCs. In unaffected NPCs, general and mitochondrial stress can be compensated by activation of effective anti-apoptotic mechanisms, whereas in SNCA-Tri cells with impaired metabolism and stress resistance, these challenges result in an accelerated induction of cell death. Considered the role of caspases in development, the altered caspase expression patterns in challenged SNCA-Tri NPCs could also affect regulation of NPC differentiation.

### Conclusion and Outlook

In “α-synucleinopathic” stem cells, α-synuclein overexpression affects cellular and metabolic plasticity, increases oxidative stress, lowers the reserve energy capacity and results in greater sensitivity for apoptosis and increased caspase activation under cellular stress. Our studies also suggest the existence of a stress threshold in NPCs, with insults and challenges (such as starvation and toxicant exposure) exceeding this threshold in SNCA-Tri NPCs at an lower concentrations and resulting in the manifestation of a “stem cell pathology” [66]. Our findings also raise new questions about the role of α-synuclein in regulation of mitochondrial activity in neuronal stem cells [67]. As the modulation of energy metabolism and metabolic signaling processes are essential for cell plasticity and cell fate decisions, our observations may have implications concerning the ability of PD-patient derived NPCs to form fully functional neuronal networks.
